# Development, Characterization and Valuable Use of Novel Dosimeter Film Based on PVA Polymer Doped Nitro Blue Tetrazolium Dye and AgNO_3_ for the Accurate Detection of Low X-ray Doses

**DOI:** 10.3390/polym13183140

**Published:** 2021-09-17

**Authors:** Saleh Alashrah, Yassine El-Ghoul, Faisal Muteb Almutairi, Mohammed Ahmed Ali Omer

**Affiliations:** 1 Department of Physics, College of Science, Qassim University, Buraidah 51452, Saudi Arabia; ashrh@qu.edu.sa; 2 Department of Chemistry, College of Science, Qassim University, Buraidah 51452, Saudi Arabia; 3 Textile Engineering Laboratory, University of Monastir, Monastir 5019, Tunisia; 4 College of Science, Al-Imam Muhammad Ibn Saud Islamic University, Riyadh 11623, Saudi Arabia; 381110204@qu.edu.sa; 5 Department of Radiologic Technology, College of Applied Medical Sciences, Qassim University, Buraidah 51452, Saudi Arabia; ma.omer@qu.edu.sa

**Keywords:** PVA nanocomposite film, silver nitrate, nitro blue tetrazolium, characterization, X-ray dosimeter, medical diagnostic radiology

## Abstract

Currently, the uncontrolled exposure of individuals to X-rays during medical examinations represents a substantial danger that threatens both medical professionals and patients. Therefore, radiation dosimetry for low X-ray doses is a very important control of radiation practice in medical diagnostic radiology. In line with this, the current study proposes a valuable dosimeter-based PVA thin film doubly doped with silver nitrate salt and nitro blue tetrazolium dye. The nanocomposite film was prepared via a simple casting method and the different processing parameters were optimized. The performance of radiation detection was evaluated according to optical, chromic, chemical and structural changes after exposure to variable low X-ray doses (0, 2, 4, 10 and 20 mGy). The different film labels exhibited an excellent stability behavior in dark and light upon 30 days of storage. The UV-Vis spectrophotometric study showed a gradual increase in the maximum absorbance as a function of the dose and the corresponding response curve confirmed this linear variation (R = 0.998). A clear structural modification was recorded via X-ray diffraction (XRD) analysis revealing the increase in crystallinity with the level of the dose received by the nanocomposite films. Microscopic surface analysis via SEM assessments revealed a significant morphological change in PVA/Ag^+^/NBT films exposed to increased radiation doses and typical dendrites growing in needle- or tree-like microstructures appeared with a high X-ray dose. Finally, the nanocomposite films before and after irradiation were evaluated via a spectrocolorimetric study and the different CIELab coordinates, the color difference, as well as the color strength, showed a linear correlation with the intensity of the applied dose. This new dosimeter design could, therefore, provide a promising and efficient alternative for prompt and accurate detection of low X-rays doses in diagnostic radiology.

## 1. Introduction

Activities using ionizing radiation are in continuous and considerable evolution. This significant development in various fields may reveal new issues in terms of radiation protection [1,2]. This is particularly the case in the medical sector with the implementation of new techniques using high energy and/or pulsed radiation for radiotherapy as IMRT and hadron therapy or low energy for radiation therapy as interventional and diagnostic radiology [3,4]. These new situations require dosimetric monitoring adapted to the complex generated fields. At the same time, dosimetric techniques have also evolved, offering, in particular, a larger choice of electronic dosimeters, in particular for neutrons, as well as a diversification of the techniques that can be used (TLD, OSL, RPL) for routine passive dosimetry [5,6,7]. 

Irradiation processing is generally investigated for different purposes, such as food irradiation, sterilization of surgical equipment, diagnostic radiology and the polymerization process [8,9,10,11,12]. It is therefore important to take an accurate measurement of the radiation dose delivered over processing. In particular, the precise measurement of low radiation doses remains a problem in view of the absence of effective and appropriate dosimeters even in a very low radiation range [13,14]. Recent advances in the field of dosimetry through the development of photographic films, Geiger-Muller and proportional counters have experienced continuous and rapid progress [15,16]. The investigation of solutions as standards, as well as the variation in oxidation states and color changes, have been extensively studied [17,18]. However, specific manipulations in addition to heavy and expensive instrumentation such as NMR, IR, UV-Vis spectrophotometer, ESR and thermoluminescence are quite necessary for the detection of changes in these solutions. [19,20]. Hence the importance and the necessity of developing simple and practical alternatives capable of detecting and quantifying radiation doses without having recourse to these additional instruments. The simplest solution was through the detection of ionizing radiations via color and shade changes [21,22]. Furthermore, having a lightweight, portable and easy-to-handle dosimeter is very required and advantageous. The most suitable choice was the use of thin films based on different polymers [23,24]. Numerous researches and publications have reported the investigation of polyamide-6, polyvinyl alcohol and polystyrene films dopped with various dyes, in the detection of various types of radiation [25,26,27].

At present, polymers and functional polymers are widely investigated in various fields [28,29,30,31,32]. The doping of various polymers with certain metal ions imparts new and improved properties to the polymer. Indeed, polymers hybridized with transition metals are considered to be versatile materials applied in many scientific fields allowing important industrial technological advances [33,34,35]. These conductive polymer composites provide the opportunity to enhance their physical, mechanical, electrical and optical properties. These composite metal chelate films based on different polymers such as poly (vinyl alcohol) (PVA), polystyrene (PS) and poly-methyl-methacrylate (PMMA) were reported with their dosimetry control applications [36,37]. The most reported metals studied as a reinforcement of polymer matrix materials, we find, are silver nitrate [38], nickel-oxide [39], bismuth germinate (Bi4Ge3O12-BGO) [40], manganese phthalocyanine [41] and ferrotitanium alloy [42]. The well-studied integration of different metals and dyes in various polymeric matrices could therefore allow practical and efficient dosimeters. Their performance is manifested by remarkable changes in physical, optical, colorimetric, structural and electrical properties after exposure to various irradiation dose levels. Plastic-films-based PVA polymers are well-reported and studied in various dosimetric applications [43,44]. PVA is the most convenient for the development of dosimetric films thanks to its low cost, water-solubility, ease of availability and possibility to incorporate a wide range of metal salts and dyes, in addition to its ability to be easily cast at moderately low temperatures. The nitro tetrazolium blue dye is previously reported by our group as an efficient indicator of low radiation doses. Its compatibility with PVA polymer is conducive to the development of a practical and effective dosimeter [45]. The investigation of AgNO_3_ with PVA thin film has demonstrated the easy formation of polymer metal chelate and the resulting film exhibited promising performance and sensitivity to low radiation doses [46].

The current study aims to take advantage of a double matrix filling using both dyes and metals to prepare an efficient composite film dosimeter based on PVA polymer and doped with silver nitrate and nitro blue tetrazolium dye. Faced with the scarcity of research on the study of hybridized polymeric dosimeters, which are effective for the detection and the quantification of low radiation doses (in mGy dose level), our versatile dosimeter represents a real challenge finding its practical importance in medical diagnostic radiology. A simple casting method will be performed to synthesize the PVA nanocomposite thin film-based nitrate chelate and NBT dye. Different parameters of the film processing were preliminarily optimized including the concentration, time and temperature of preparation. Different X-rays doses (0, 2, 4, 10 and 20 mGy) were then applied to the synthesized films. Afterward, chemical, structural, physical and morphological characterizations via UV, XRD and SEM analysis were performed on the raw and exposed films. In addition, the colorimetric properties of the irradiated films were evaluated in order to assess the efficiency and the sensitivity performance of these developed dosimeters. Furthermore, the determination of the various curves illustrating the responses, resulting from the various mentioned characterizations, according to the increasing doses applied to PVA/Ag^+^/NBT nanocomposite films was carried out to evaluate the performance of the dosimeter in the field of X-ray diagnostic radiology. 

## 2. Experiment

### 2.1. Materials

The silver nitrate (AgNO_3_, Mw = 169.87 g/mol) and nitro blue tetrazolium chloride (C_40_H_30_ Cl_2_N_10_O_6_·H_2_O·CH_4_O; MW = 867.70) investigated as matrix reinforcements for the different dosimetric films, as well as the polyvinyl alcohol polymer (PVA, M_W_= 85–124 kDa with a hydrolysis degree of 85–90%), were acquired from Sigma Aldrich (St. Louis, USA). Ultrapure water (Milli-Q^®^ Direct, Darmstadt, Germany), was used for the preparation of the film nanocomposites. All reagents employed were of analytical grade and were used in the various experiments without further purification.

### 2.2. Characterization of Film Nanocomposites

The synthesized nanocomposite films were characterized via different techniques and the comparison between the raw and irradiated films at various doses was beneficial for the evaluation of the efficiency of the films as low X-ray radiation dosimeters. 

FTIR analysis via the attenuated total reflection (ATR) method was carried out for the determination of various spectra of raw and irradiated films using an infrared spectrophotometer (Agilent Technologies/Gladi-ATR, Santa Clara, CA, USA). A range scale varied from 4000 to 400 cm^−1^ and a resolution of 4 cm^−1^ were fixed for the measurement of different infrared spectra. 

A UV–vis spectrophotometer (Shimadzu, UV-2501PC, Kyoto, Japan) was investigated for the measurement of the absorption curves of different film labels. The measurements were carried out on a scale of wavelength varying from 200 to 700 nm.

An X-ray Diffractometer (PW 1720 Philips) was used to obtain the X-ray diffraction patterns. The samples were scanned at the 2θ angle varying from 10° to 90°. The step width was 0.01°. The operating voltage and current intensity were 40 kV and 40 mA, respectively.

The mechanical study was performed using a Universal Testing Machine (Zwick Testing Machine Ltd., Leomister, UK). Film samples were cut as 5 mm x 40 mm. A crosshead speed of 5 mm/min was set throughout the experiment. The measurements were carried out according to the standard ASTM D638 at 22 °C with 65% relative humidity. Each measurement was an average of five replicates. 

The morphological study of the surface of raw and X-ray exposed films was accomplished via SEM analysis. Different SEM micrographs were obtained using Scanning Electron Microscopy (JEOL JSM-5400 LV, JEOL Ltd., Akishima, Japan). A 5 kV acceleration voltage was selected during measurements. Magnifications were varied from 100 to 2000×. In order to improve surface conductivity, all samples were subjected to a surface coating of a thin layer of Au before analysis.

The evaluation of colorimetric characteristics of raw and irradiated films was carried out using a spectrocolorimeter instrument (3NH-YD5010) operating in the visible spectrum at 39 wavelengths in a wavelength range varying from 380 to 780 nm. The color strength (K/S) values were measured using the equation of Kubelka–Munk Equation (1) [47].

(1) $${\left(\frac{K}{S}\right)}_{\lambda \;}=\frac{{\left(1-{\;R}_{\lambda }\right)}^{2}}{2\times {\;R}_{\lambda }}$$

where K is the absorption coefficient, S is the scattering factor and R_λ_ represents the spectral reflectance of the colored film at λmax [48].

The different CIELab color coordinates of PVA/Ag/NBT nanocomposite films (L*, a*, b*) and the ΔΕ variation representing the color total difference were measured under a standard observer of 10° and the daylight standard illuminant D_65_. L* represents the lightness coordinate, a* and b* describe the redness-greenness and the yellowness-blueness axis, respectively [49,50].

The ΔΕ values were calculated as a function of the various measured color coordinates as following (Equation (2)).

(2) $$\Delta E=\sqrt{{\left(L^{*}-L_{0}\right)}^{2}+{\left(a^{*}-a_{0}\right)}^{2}+{\left(b^{*}-b_{0}\right)}^{2}}$$

where (L*, a*, b*) and (L_0_, a_0_, b_0_) are the measured CIELab coordinates for the raw and irradiated PVA/Ag/NBT films, respectively. The average limit of the distance ΔΕ between two CIELab coordinates is the value above which the human eye cannot differentiate. Many limits of ΔΕ depending mainly on color saturation have already been reported in various research works [51,52].

### 2.3. Preparation of PVA/NBT Nanocomposite Films

The films-based PVA polymers mixed with various amounts of NBT and silver nitrate fillers were developed following a previously reported casting method in our laboratory [45]. A 5% solution of PVA dissolved in distilled water was prepared. The bulk solution was then heated gently, using a heater instrument with controlled temperature. The preparation was kept under continuous stirring at a constant temperature of 80 °C. After 2 h, the polymer solution was cooled, to an ambient temperature and two prepared solutions of NBT (0.04% dissolved in 10 mL of ethanol) and AGgNO_3_ (0.04% dissolved in 10 mL of distilled water) were poured in the viscous clear solution of PVA. After 1 h of stirring without heating, 20 mL of the solution was poured in glass petri dishes. The dishes were then set to dry in dark (to avoid direct exposure to light) at room temperature for 3 days. The thickness of peeled films was then measured using a thickness numerical instrument. The mean value of the thickness of films was around 100 μm. The nanocomposite films were finally cut into small pieces of 2 cm sides and stored in black envelopes away from any light to avoid their possible deterioration and to achieve thermal equilibrium before their next use. 

A digital X-ray fluoroscopic machine (GE, healthcare, model Al01CII, Chicago, USA) was used for the irradiation of different films with variable selected doses. This fluoroscopic device was programmed by adjusting the various technical exposure factors (kVp = 60, mAs = 16 and at 100 cm) thus producing the desired exposure doses of x, y, z mGy. The system was calibrated and verified via an ionization chamber to generate the appropriate doses.

## 3. Results and Discussion

### 3.1. Post-Irradiation Stability

**Figure 1:**
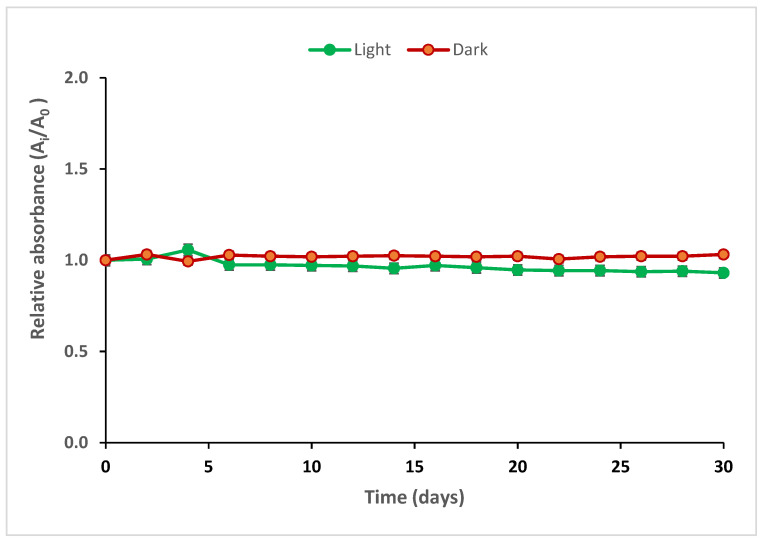
Post-irradiation stability of PVA/Ag^+^/NBT films after exposure to an X-ray dose of 20 mGy.

PVA/Ag^+^/NBT prepared films and treated with a dose of 20 mGy were stowed in light and dark at an ambient temperature. The storage of different films was for 30 days. The absorbance at 460nm was determined during this storage at varying time intervals. The variation of the maximum absorbance of the irradiated nanocomposite films measured as a function of the storage period in light and in dark is presented in Figure 1.

Figure 1 shows the excellent stability of the different films stored at variable times. Irradiated nanocomposite films exhibited good stability both in light and dark storage conditions for 30 days.

This confirmed stability of the PVA/Ag^+^/NBT nanocomposite films is important for practical dosimeters and more effective than other reported film dosimeters consisting only of PVA and silver nitrate or the PVA/NBT in solution or gel form [53,54]. This is due to the rigidity of the compact form of the casted film and the good compatibility between PVA, the organic dye and the metal chelate. The homogenous mixture of these three protagonists provided the nanocomposite film with excellent crystalline properties. 

### 3.2. Direct Perception of Color Change after Irradiation

**Figure 2:**
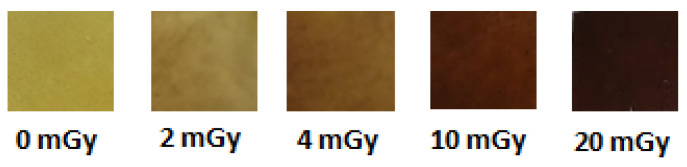
Direct perceived color change of PVA/Ag^+^/NBT films irradiated with different X-ray doses.

A significant color deviation was directly perceived with eyes after irradiation of the different nanocomposite thin films. Figure 2, reveals a gradual color change according to the intensity of the X-ray dose. The color deviation gradually evolves with the dose from a yellow for unirradiated film to a dark yellowish brown shade. The color modification is related to the reduction of NBT2+ to mono-formazan (MF+) then to a stable hydrophobic di-formazan (DF) structure [55] and also due to the reduction of silver ions in the PVA metal chelate complex into metallic silver Ag^0^ [56]. The two PVA matrix fillers exhibited the ability to induce a color change after irradiation. These changes in shade detected following irradiations of very low doses prove well the high sensitivity of these developed dosimeters and thus they are effective for the precise control of the doses in diagnostic radiology.

### 3.3. UV/Visible Absorption Spectra

**Figure 3:**
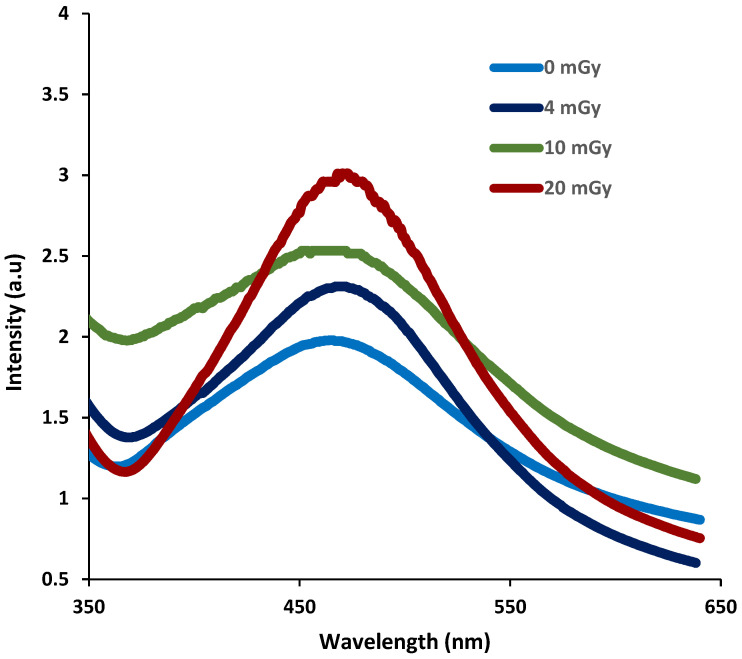
UV-Vis spectra of PVA/Ag^+^/NBT nanocomposite films before and after exposure to increased X-ray doses.

The absorption spectra of irradiated PVA/Ag^+^/NBT films were measured in the wavelength range of 200–800 nm. The raw un-irradiated film was considered a reference. The different absorption spectra of nanocomposite films were recorded at variable X-ray doses. The film gradually turns dark yellowish-brown after X-ray irradiation. Absorption spectra of different PVA/Ag^+^/NBT films show in Figure 3 a wide peak centered at 462 nm. The absorption intensity of the peaks at this wavelength increased progressively according to increasing X-ray doses. This gradual increase shows, throughout, that the trends of the curves are related to the reduction of NBT2^+^ to mono-formazan (MF^+^) then to a stable hydrophobic di-formazan (DF) structure [57,58] and also due to the reduction of silver ions in the PVA metal chelate complex into metallic silver Ag^0^ [59,60] under the effect of the applied irradiation. This change in absorption behavior in the visible region after irradiation is manifested also by the modification in perceived color. Indeed, under the effect of radiation the color undergoes a deviation from yellow to dark yellowish-brown after reduction of NBT [57,58] and from yellow to dark green following the reduction of silver [61]. Moreover, the maximum absorption of the different spectra gradually shifted, with the increase in X-ray doses, from 462 to 472 nm (with a dose of 20 mGy). This was probably due to the more prominent reduction of both Ag+ ions providing Ag^0^ and formazan for the NBT thus allowing the di-formazan product.

### 3.4. Response Curve

**Figure 4:**
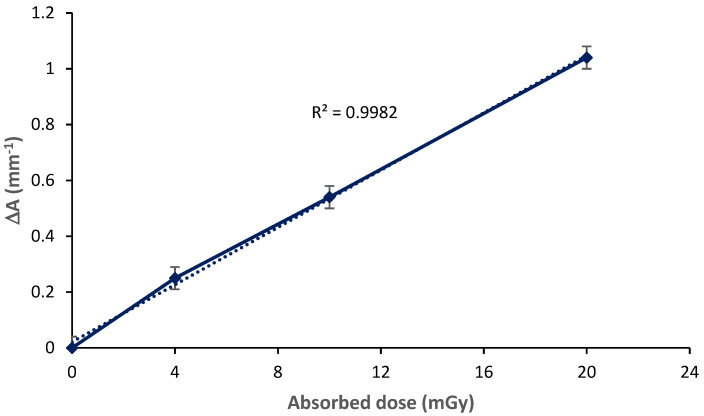
Dose–response curve based on UV-Vis absorption capacities at λmax for PVA/Ag^+^/NBT films exposed at various X-ray doses.

Figure 4 shows the variation in absorbance capacity as a function of the increase in the applied X-ray dose. This dose response curve revealed a perfect correlation with the intensity of the dose and the variation is practically linear presenting a coefficient of linear regression R = 0.998. This perfect correlation is very significant in the application of PVA/Ag^+^/ NBT thin films as effective dosimeters in the detection and quantification of low X-ray doses.

### 3.5. X-ray Diffraction Study

**Figure 5:**
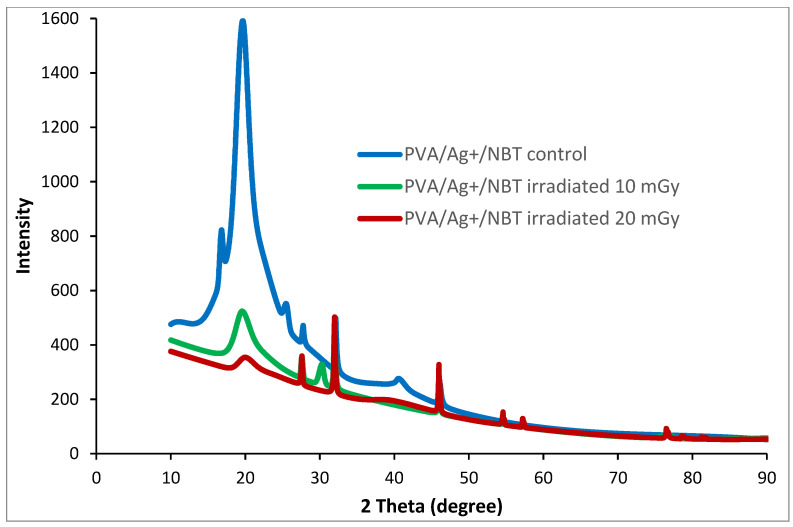
XRD patterns of control and irradiated PVA/Ag^+^/NBT nanocomposite films.

The X-ray exposure could generate some structural changes that are detectable by XRD analysis. XRD characterization was conducted to assess the different structural modifications and rearrangements revealed in the XRD diffractograms due to X-radiation treatment of the nanocomposite films with increased doses. As shown in Figure 5, the different prominent peaks in XRD patterns were well in agreement with the literature values [62]. XRD diffractogram of the unirradiated sample divulges the presence of a characteristic peak centered at 2θ = 16.6°, which was ascribed to the diffraction plane $(00\bar{1}$) of PVA. This peak, referring to the amorphous state, disappeared after irradiation treatment. Furthermore, XRD patterns show a principal peak at 2θ = 19.64◦ which is assigned to the diffraction from a mixture of planes (101) and ($10\bar{1}$) [63]. This peak shifts to the higher angle side from control to irradiated films. In addition, we noticed the decrease in the intensity of this important diffraction peak with the increase in the X-ray irradiation dose implying the decrease in the crystallinity of treated films. This result confirmed the presence of an obvious structural rearrangement in the polymer matrix varying with the intensity of the applied dose. 

### 3.6. Mechanical Characterization

**Figure 6:**
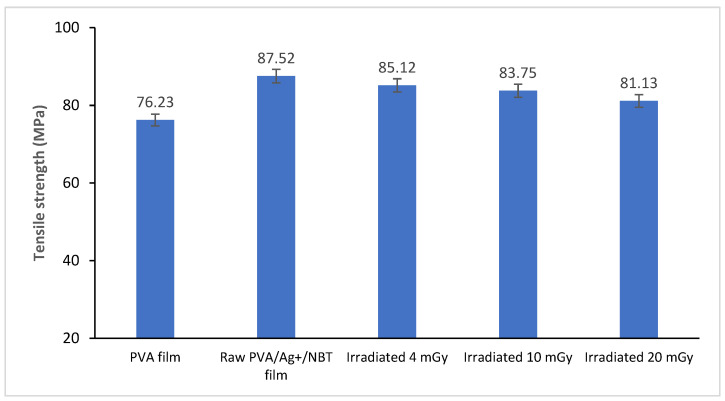
Tensile strength variation of raw PVA film, control and irradiated PVA/Ag^+^/NBT nanocomposite films with increased X-ray doses.

Figure 6 shows the variation of the tensile strength of the PVA film, as well as untreated and irradiated PVA/Ag^+^/NBT nanocomposite films. We noticed a relatively significant increase in tensile strength after the incorporation of NBT and Ag into the matrix of the nanocomposite film. Therefore, this confirmed the absence of phase separation on the doped film, thus indicating good compatibility between NBT, silver and PVA, which improved the tear-resistance of the made composite film. This phenomenon can be caused by the presence of hydrogen and coordination bonds which enhance the intermolecular forces between the NBT, the silver and the base PVA matrix and thus improve the tensile strength of the PVA blend film [64]. With increased X-ray doses, the tensile strength decreased slightly. This confirmed the reduction of the crystalline structure after irradiation (confirmed from XRD analysis). The decrease in mechanical properties suggests that irradiation induces structural rearrangement and probably the prevalence of intermolecular and chain scission processes. 

### 3.7. SEM Morphological Analysis

**Figure 7:**
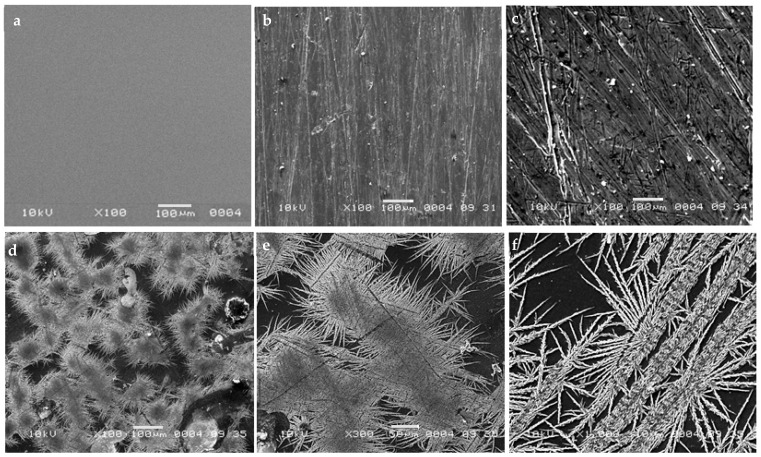
SEM micrographs of PVA/Ag^+^/NBT control film (**a**) and irradiated PVA/Ag^+^/NBT films with 4 mGy (**b**), 10 mGy (**c**) and 20 mGy with different magnifications (**d**–**f**).

Control and treated samples with various X-ray doses were characterized by SEM analysis to assess the effect of different radiation doses on the surface morphology and microstructure of exposed nanocomposite films. Concerning the non-irradiated film, Figure 7a shows a uniform, smooth homogeneous and compact microstructure without any phase separation thus showing the excellent compatibility of the various protagonists of the PVA matrix [65]. We notice after radiation treatment a clear modification of the morphology of the exposed films with the appearance of some fluke-like structures and many surface stripes (Figure 7b,c). The surface degradation is all the more pronounced and intense with the increased X-ray doses. An alteration manifested by a significant microstructural change is illustrated by regular forms of cracks extending in all directions (Figure 7d,e,f). These micrographs at increased magnifications show dendritic characters with a clear main trunk and side branches. Dendrite growth occurs as a result of the reduction of metal ions dissolved in the nanocomposite film, thereby growing in needle- or tree-like microstructures [66]. This form of degradation detected in the film after irradiation could therefore be specific to our nanocomposite film, thus offering more specificity and performance to this dosimeter designed for low doses of X-rays.

### 3.8. Colorimetric Study

The color deviation in intensity or shade after irradiation is a crucial property of an effective dosimeter. The performance becomes more practical when the color change varies with dose and is easily perceived by the human eye. A spectrocolorimetric study was carried out to assess and quantify the color variation of the PVA/Ag^+^/NBT nanocomposite films after their irradiation with increased low X-ray doses. The mean deviation of the CIELab coordinates L*, a*, b*, the total color difference (ΔE) and the color strength (K/S) evaluations for the control and the irradiated PVA/Ag^+^/NBT prepared films are presented in Table 1.

**Figure 8:**
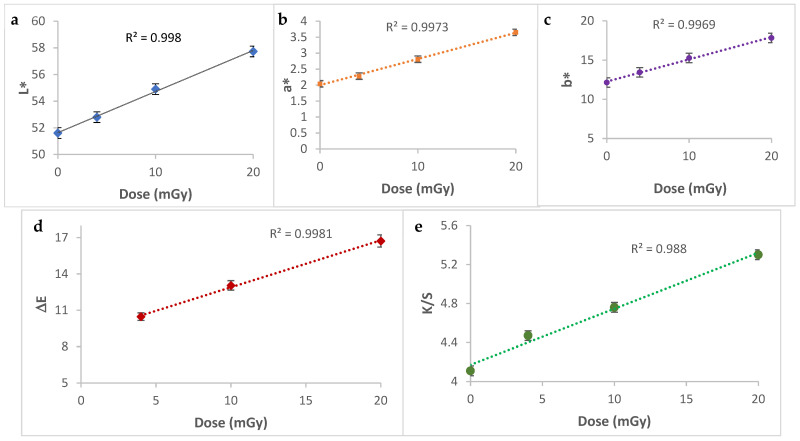
CIELab color parameters (L*, a* and b*) (**a**–**c**), total color difference (ΔE) (**d**) and color strength (K/S) (**e**) measurements for PVA/Ag^+^/NBT films irradiated from 4 to 20 mGy.

The different measurements of the three color coordinates in the CIELab space, L*, a* and b* according to the X-ray dose irradiating the various treated films, are presented in Figure 8a–c. The tendency of the lightness (L*) curve decreases linearly with increasing treatment dose, this means that the color becomes darker and darker. However, the Cartesian color coordinates a*, b* increased with the intensity of the applied dose, this means that the color of the films is more reddish and bluish. The variations measured via the spectrocolorimeter clearly show a perfect correlation between each color parameter and the intensity of the applied dose. The linear dependence of the different color coordinates according to the increased applied radiation dose allows each color’s coordinate a*, b* and L* to be investigated separately as a dosimetric index for low dose radiation varying from 0 to 20 mGy. Furthermore, Figure 8d shows the perfect increase of the ΔE (representing the total color deviation) with the intensity of X-ray treatment doses. This total color difference for all the doses is much higher than 1 (from 3.5 to 9.5) so the color change is clearly perceptible with the human eye. The ΔE values vary linearly with increased doses applied to the different samples. The same tendency was observed for K/S (color strength). This important color parameter increased quasi-linearly with higher radiation doses (Figure 8e). The spectrocolorimetry study revealed well the efficiency of the designed nanocomposite PVA/Ag^+^/NBT thin film as a performance irradiation indicator. The direct perceived change in color reflects well the potential of this dosimetric system to control low radiation in diagnostic radiology. 

## 4. Conclusions

The accurate evaluation of the X-ray dose to the patient is an essential aspect of the control process in X-ray diagnostic radiology. Therefore, it is a crucial necessity to develop an effective dosimetric system for the accurate evaluation and quantification of the X-radiation in diagnostic radiology examinations. In this study, we developed a new designed dosimetric film-based PVA polymer doped with both silver nitrate salt and NBT dye. The performance of the nanocomposite film has been confirmed via the different applied characterizations after increased radiation treatments. Indeed, the absorption capacity varied linearly with the intensity of the X-ray doses. This was due to the reduction of NBT^2+^ to mono-formazan and di-formazan form and the reduction in the same time of the silver ion Ag^+^ in the polymer metal chelate to Ag^0^, under the effect of the X-ray treatment. XRD assessment via the investigation of the different patterns revealed significant structural modification and crystallinity change after various radiation exposures. SEM analysis showed a clear morphological modification of PVA/Ag^+^/NBT thin films after X-ray treatment depending on the applied dose, confirming thus the high sensitivity of the dosimetry films upon very low doses. Furthermore, the different developed thin films exhibited excellent post-irradiation stability in light and dark from 5 to 30 days after X-ray exposure. The spectrocolorimetric study was the most important in view of the practical impact of a color change after irradiation exposure. All color parameters showed linear quantitative (measured coordinates) and qualitative (direct perception of color) variation with increasing treatment dose. Moreover, the total color difference and the color strength were also shown to be linearly increased with augmented radiation doses. The various characterization assessments confirmed the excellent radiation detection via the designed nanocomposite films exposed to very low radiation doses (in the mGy scale). The developed polymeric nanocomposite film has shown practical efficiency as a new potential irradiation indicator for routine diagnostic control processes. 

## Figures and Tables

**Table 1:** CIELab coordinates (L*, a*, b*), ΔE and K/S measurements for control and irradiated PVA/Ag^+^/NBT films from 4 to 20 mGy.

Radiation Dose	L*	a*	b*	ΔE	K/S
0	51.61	2.04	12.14	-	4.11
4	52.80	2.28	13.44	10.47	4.47
10	54.90	2.81	15.27	13.05	4.76
20	57.73	3.65	17.83	16.73	5.30
